# Structural analysis of a calix[4]arene-based Platonic Micelle

**DOI:** 10.1038/s41598-018-38280-1

**Published:** 2019-02-13

**Authors:** Efstratios Mylonas, Naoto Yagi, Shota Fujii, Kodai Ikesue, Tomoya Ueda, Hideaki Moriyama, Yusuke Sanada, Kazuya Uezu, Kazuo Sakurai, Tadashi Okobira

**Affiliations:** 1 Japan Synchrotron Radiation Research Institute (JASRI/SPring-8), 1-1-1, Kouto, Sayo-cho, Sayo-gun, Hyogo 679-5198 Japan; 2 Department of Chemical Processes and Environments, Faculty of Environmental Engineering, The University of Kitakyushu, 1-1, Hibikino, Wakamatsu-ku, Kitakyushu 808-0135 Japan; 3 Present Address: Institute of Molecular Biology and Biotechnology, 70013 Heraklion, Crete Greece; 4 Department of Creative Engineering, National Institute of Technology, Ariake College 150 Higashihagio, Omuta, Fukuoka 836-8585 Japan; 5 Structural Materials Science Laboratory SPring-8 Center, RIKEN Harima Institute Research, 1-1-1 Kouto, Sayo, Sayo, Hyogo 679-5148 Japan

## Abstract

We have recently introduced the concept of “Platonic micelles”, the preference of spherical micelles to specific aggregation numbers mostly coinciding with the number of faces of platonic solids. This effect was observed on bulky, mostly calix[4]arene-based surfactant systems with small aggregation numbers. The preferred aggregation numbers result in better sphere coverage, highliting the packing and the “protection” of hydrophobic cores from the aqueous solvent as the most important factor for this preference. In the present study we further explore the interactions that drive the packing of the highly charged PACaL3 surfactant into highly symmetrical hexameric micelles. We performed a series of molecular dynamics simulations that yielded a large set of structures and an ensemble in good agreement with the experimental Small Angle X-ray Scattering data was selected. The geometry and the rigidity of the calix[4]arene group with proper tail length and headgroup volume are the driving forces for the high symmetry and monodispersity of the micelle. The charge of the headgroups is mainly responsible for inhibiting the formation of higher order structures. Sodium, shown to be important for the stability of the micelle, is not directly interacting with the micelle implying that the calix[4]arene ring is a C2ν symmetry conformation.

## Introduction

Micelles formed by amphiphilic molecules, i.e. lipids or surfactants, have always attracted scientific interest because of their ability to self assemble and form interesting three-dimensional structures. However, recent advances in the field, not least because of their potential as non-viral drug or gene delivery systems, have led to the synthesis of molecules that exhibit previously unreported and unusual properties. The interactions that lead to the formation of these structures is not dissimilar to the forces that lead to the formation of “traditional” micelles or, even, the folding of proteins and nucleic acids. The interplay between hydrophobic, van der Waals, electrostatic, π-π interactions and steric constraints and their relative importance is specific to the molecule being investigated.

One of the most interesting properties of some micelles is that depending on the environmental conditions they are able to drastically change their structure and in fact one can take advantage of this environmental response and manipulate the shape of the micelles formed^1^. Moreover, there is a an ever-increasing numbers of newly synthesized, often calix[4]arene-based, amphiphiles, that assemble into completely uniform and structurally precise micelles consisting of asmall number of molecules^2–5^. Molecular dynamics (MD) simulations can prove useful in identifying the interactions that lead to the formation and the properties of these micelles.

The combination of Small Angle X-ray Scattering (SAXS) data with the results of MD simulations has been employed for the structural analysis of proteins with intrinsic flexibility^6^.

Though SAXS data is ambiguous, i.e. different shapes can produce the same scattering pattern, when used on a physically plausible atomic-detail model it is actually an excellent method when one needs to choose between structures that appear otherwise quite similar. Likewise MD can be extremely valuable, if not as a model representing molecule or supramolecular assembly movement, as a tool for exploring the conformational space assuming that a reasonable initial model is provided. The SAXS data can then be used for the experimental validation of the produced trajectory. Thus, the combination of these experimental and computational techniques can provide structural information that would otherwise be inaccessible.

**Figure 1:**
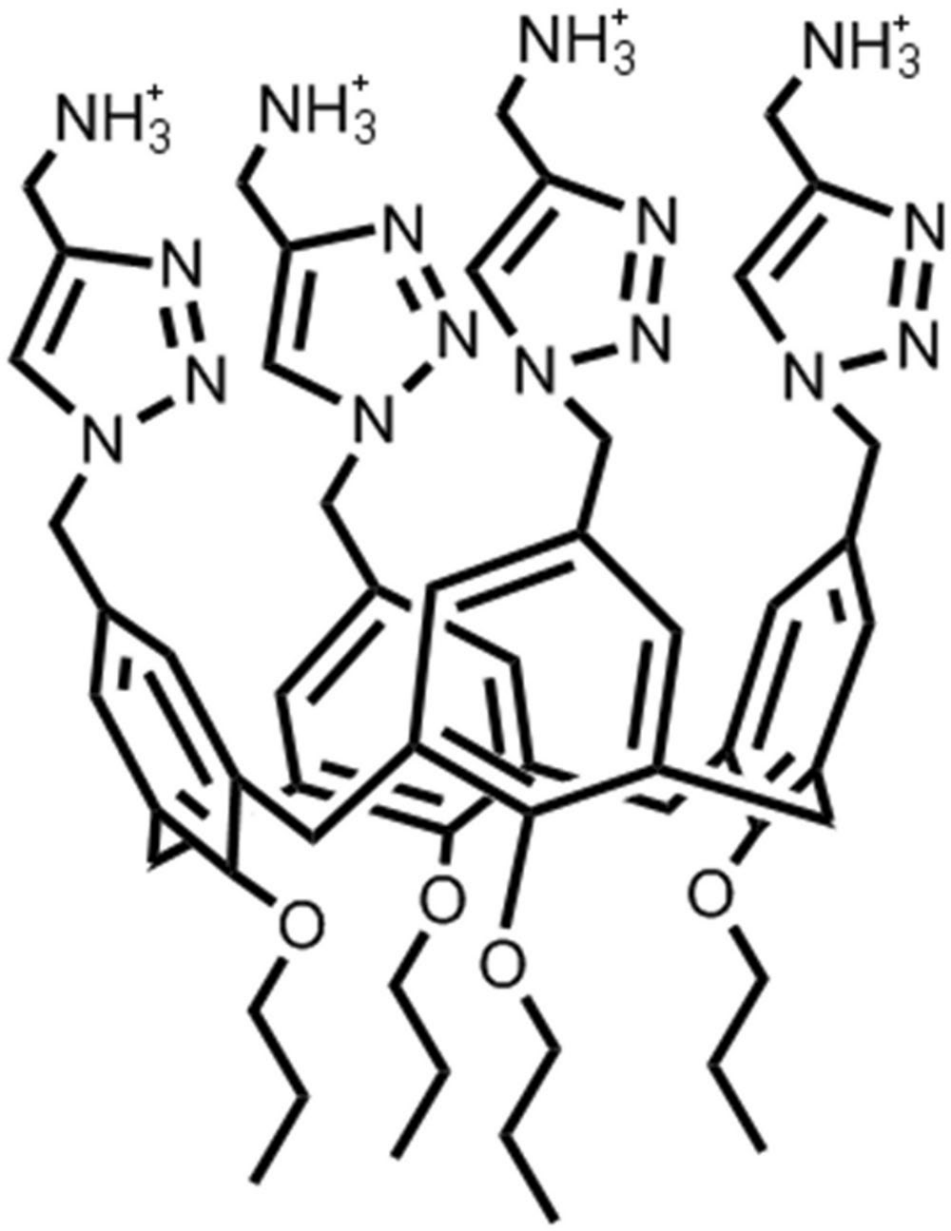
Scheme of the PACaL3 surfactant showing the 1,2,3-triazole rings and charged amine groups of the headgroups and the propyl tails connected to a calix[4]arene ring.

A variety of different surfactants can form completely uniform and structurally precise monodisperse micelles, depending on the tail length, hydrophilic head volume, granted that they have sufficiently small aggregation numbers^4^. These numbers are good solutions to the Tammes problem, i.e. provide good coverage of a sphere with specific numbers of spherical caps. Because these numbers partly coincide with the number of faces on platonic solids we named them “Platonic Micelles”. The present communication will further explore the low pH micelles formed by the PACaL3 surfactant (Fig. 1) that we examined in a previous study^5^ (termed CaL[4]C3 before). This surfactant forms very monodisperse spherical micelles (see Supplementary Information Fig. S1 for the Kratky^7^ and Porod^8^ plots) at low pH and at higher pH values it transforms to cylinders due to the neutralization of the charge of the amine headgroups. Primary SAXS analysis, as well as fitting to a simple core-shell model were conducted in our previous study. We have also shown by a couple of experimental techniques that the spherical micelles have a well-defined aggregation number of six and that the existence of sodium chloride is important for the stability and monodispersity of the micelle. With the structural information provided by the SAXS data, we employ these findings to the analysis of structural models produced by MD simulations and explain them on the basis of the interactions that facilitate the formation and monodispersity of these micelles.

## Materials and Methods

The synthesis of the PACaL3 molecule, the SAXS measurements and primary data reduction were carried out as shown before^5^. Only the spherical micelles at low pH were considered in our calculations and comparison with the SAXS data.

Rigid body model calculations of the PACaL3 micelles were performed by the program SASREF^9^ with the original conformation of the molecules calculated with MOPAC^10^. Six molecules of PACaL3 were used for the rigid body model and distance restraints between molecules were introduced to ensure that the tail groups point to the inside of the structure.

All MD simulations were carried out using the Amber 11 program suite^11^ on a CUDA workstation with the parameters of the General Amber Force Field (GAFF)^12^. The initial geometry of the molecule was optimized with the HF/6–311 G(d,p) basis set with Gaussian 09^13^. The partial charges of the atoms were determined with the RESP ESP charge Derive (R.E.D.) tools III.4^14^. Six PACaL3 molecules were initially arranged in cubic symmetry with each molecule occupying one face of a cube. The initial arrangement was as tight as possible without steric clashes between the molecules. The molecules were immersed in a TIP3P box with periodic boundary conditions and all simulations were performed in explicit solvent at 300 K and constant pressure of 1 atm after an initial minimization and stepwise heating in constant volume and constraints. The possibility of implicit solvent simulations was explored but the instability of the micelles in this environment made it an unviable option (the molecules dissociate shortly after starting the simulations). Simulations up to 320 ns were performed with timesteps of 1 or 2 fs.

The SAXS patterns of structures from snapshots taken at regular intervals during the simulations were computed with CRYSOL^15^. While it was possible to use the water molecules closest to the micelles as the hydration shell, we opted for CRYSOL’s built-in facility to account for the scattering of the hydration shell. It has the advantage of being computationally much cheaper, and because of the difficulty and uncertainty of determining which individual water molecules belong to the hydration shell it leads to somewhat better results, i.e. better fit to the experimental data. The best agreement with the experimental data was achieved when we used up to the second hydration layer water molecules in the case of explicit water molecules from MD. Similarly, a large value of the contrast of the hydration shell (0.06–0.075) is generally required by CRYSOL for better agreement with the experimental data.

The visualization and analysis of the trajectories as well as the calculation of the pair distribution functions were performed with tools within the VMD software^16^ and CPPTRAJ^17^. The Jaccard index was calculated with a C# application, developed in-house.

## Results

### Rigid body model calculations

A first aproximation of the structure of the micelle was obtained from rigid body modeling. Six copies of a low energy model of PACaL3 calculated with MOPAC^10^ were used to create a rigid body model with SASREF^9^. The rigid body model is created by moving the individual molecules around trying to find the configuration that best fits the experimental data. The actual properties of the molecules are not accounted for. The simulated annealing procedure is guided by the fit to the experimental SAXS data with some penalties implemented in the scoring function to avoid steric clashes and disconnectivity between the different subunits. Since we know that micelles have the carbohydrate groups hidden in the center of the molecules we added distance restraints between the tips of the tail groups in order to have them oriented correctly.

**Figure 2:**
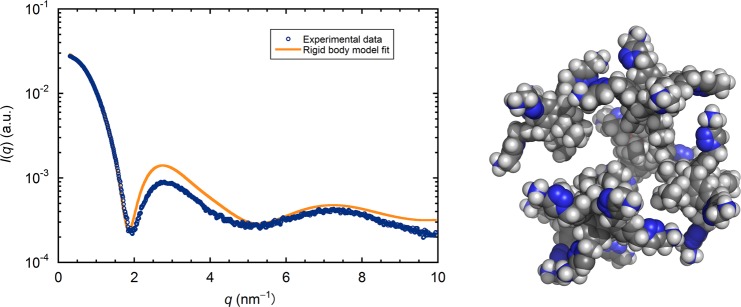
Experimental SAXS data of the low pH PACaL3 micelles (blue circles) and comparison to the calculated scattering of the rigid body model shown on the right.

The fit to the experimental SAXS data is shown in Fig. 2 together with the produced model. While there are differences from the experimental data, the calculated pattern follows very faithfully its peaks and troughs, considering the high resolution and low noise of the data. The features of the calculated pattern are somewhat sharper which is to be expected since the actual micelle should experience some dynamic fluctuations that are not reflected on the rigid body. On the other hand, the model does not look very realistic. The distance between the molecules is too great and it looks like there is a hole in the middle of the structure. The most likely explanation for this result is that the individual molecules cannot change their initial conformation since they are treated as rigid bodies. It is impossible to bring them too close even if we imposed stronger restraints because in that case there would be steric clashes between the molecules.

It is evident that for this kind of system rigid body modeling is not very appropriate since it does not take into account the physical properties of the molecules and it does not allow for the plasticity of the molecules required to make a more compact structure. For this purpose, we proceeded to perform molecular dynamics simulations constructing initial structures inspired by the rigid body models.

### MD simulations and correlation with the SAXS data

Production runs of Molecular dynamics simulations up to 320 ns in explicit solvent were carried out. Variation of *R*_g_ and root mean square deviation (RMSD) over the course of a 320 ns simulation and radial distribution function (RDF) of waters are shown in Supplementary Information Figs S2, S3 and S4 respectively. Comparison of the *R*_g_ variations over shorter (20 ns) simulations are shown in Supplementary Information Fig. S5. While it is usually advisable to run MD simulations for as long as possible in order to allow the system to reach an equilibrium, in our case this proved counterproductive since no significant change in the energy of the system was observed (data not shown). Running the simulation of the micelle for too long resulted in the deformation of the micelle and occasionally to the dissociation of the micelle. On the rare occasion that one of the molecules escapes the micelles it is effectively impossible to return and the remaining pentameric micelle is more unstable than the original hexameric one. Moreover comparison with the experimental SAXS data further reinforced the idea that it was counterproductive to run very long simulations.

The fit of the scattering pattern calculated for an average over all MD structures to the experimental SAXS pattern is not satisfactory and in fact the calculated scattering pattern fails to reproduce even the first sharp minimum observed in the experimental data. This denotes that the full trajectory of the MD simulation is not a good representation of the conformational space explored by our micelle.

Nevertheless, the snapshots of an MD simulation are valuable as a large pool of possible models in a variety of different conformations. By comparing with the experimental data we are able to select among these different conformations the ones that better represent the actual structure in solution.

Micelles are dynamic assemblies that exhibit substantial movement. Because of this, a micelle is actually constantly changing its structure and SAXS patterns are an averaging of all the different conformations the micelle adopts.

First, the Jaccard index^18^ was adopted for comparing the similarity of the scattering profiles between MD simulations and experimental SAXS and finding the most plausible MD models. The Jaccard index is a measure of the goodness of the fit of experimental to calculated SAXS data. It can be understood as an error-independent normalized inverse of the χ^2^ that is most commonly used, with the advantage of producing values ranging only from 0 to 1. It is defined as the quotient between the intersection and the union of the pairwise compared variables among two objects.

$${J}_{ind.}=\frac{{\sum }_{i=0}^{N}[\mathrm{log}\,I({q}_{i})\times \,\mathrm{log}\,{I}_{c}({q}_{i})]}{{\sum }_{i=0}^{N}{[\mathrm{log}I({q}_{i})]}^{2}+{\sum }_{i=0}^{N}{[\mathrm{log}{I}_{c}({q}_{i})]}^{2}-{\sum }_{i=0}^{N}[\mathrm{log}\,I({q}_{i})\times \,\mathrm{log}\,{I}_{c}({q}_{i})]}$$

here, *I*(*q*_*i*_) and *I*_*c*_(*q*_*i*_) are the observed and calculated scattering intensities at the same *q*_*i*_. In our case, the closer the value of the Jaccard index is to 1, the better the fit of the calculated to the experimental SAXS data.

**Figure 3:**
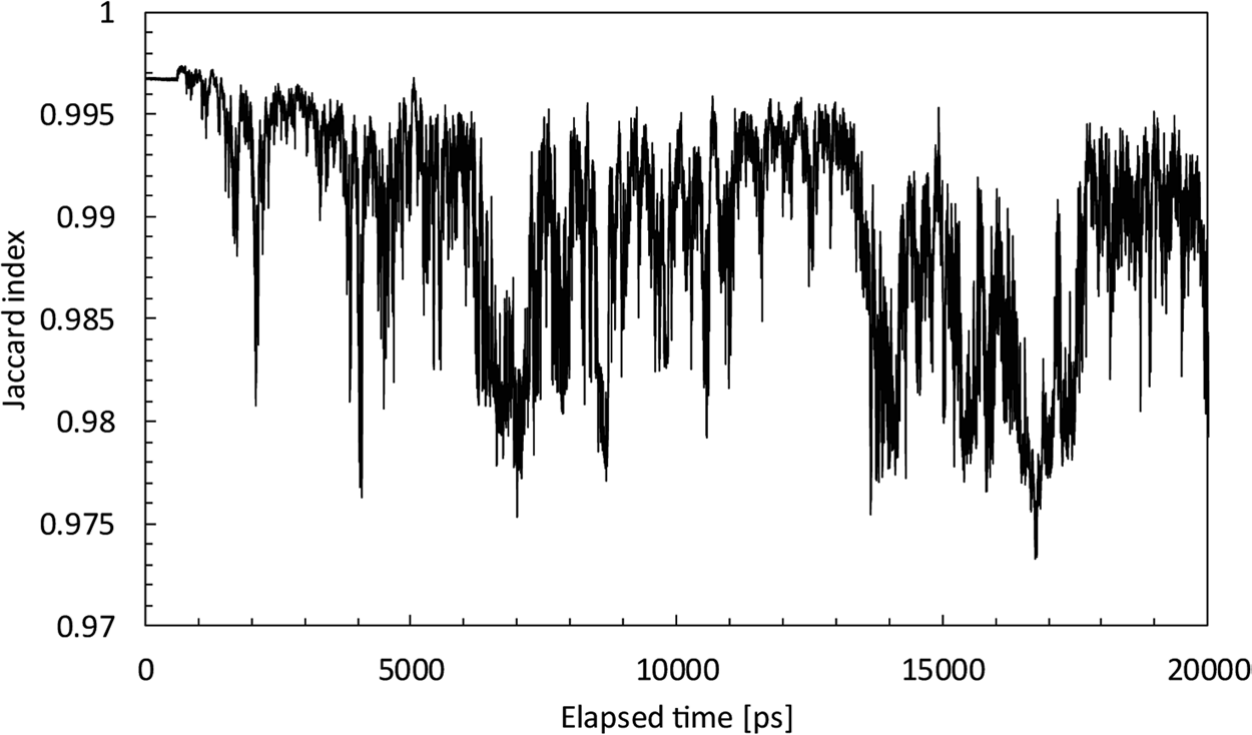
Temporal change over the course of MD simulations of the Jaccard index between the MD structures and the SAXS experimental pattern.

**Figure 4:**
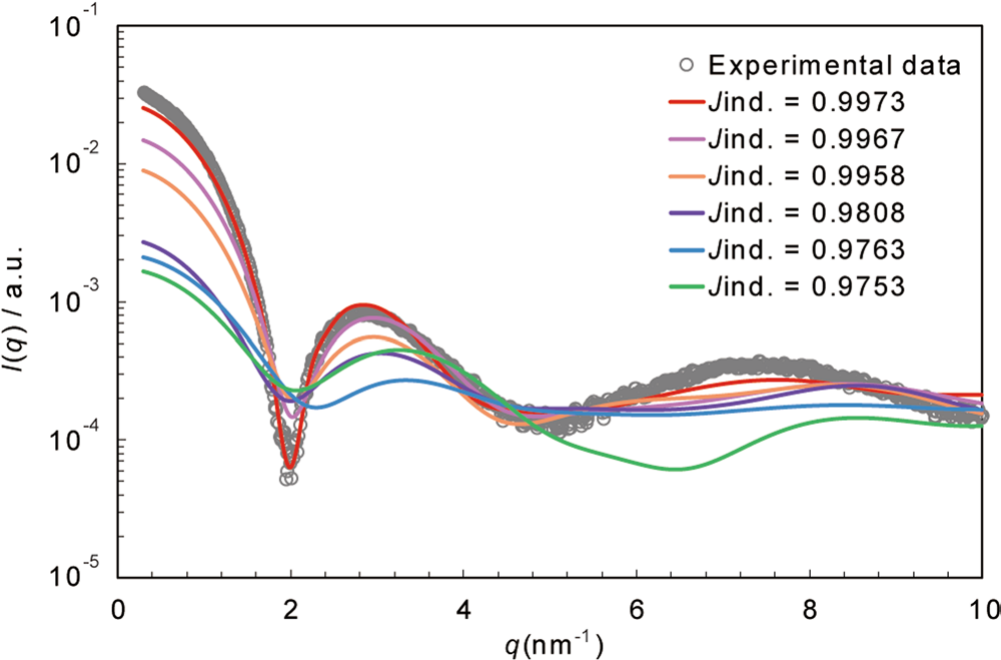
Experimental SAXS data of the spherical PACaL3 micelle (gray circles) and fitting results of the MD structures (colored).

Figure 3 shows the temporal change of Jaccard index of the fit of the experimental SAXS data to structures retrieved over the course of MD simulation. In our simulation, the structure of the PACaL3 micelle varies greatly due to thermal motion, so Jaccard index changes significantly. Interestingly, the starting, hexagonal packing structure, once collapsed by thermal fluctuations, occassionally re-emerges over the course of the simulation. Most structures with high Jaccard index values (above 0.996) in Fig. 4 form hexagonal packing structures.

**Figure 5:**
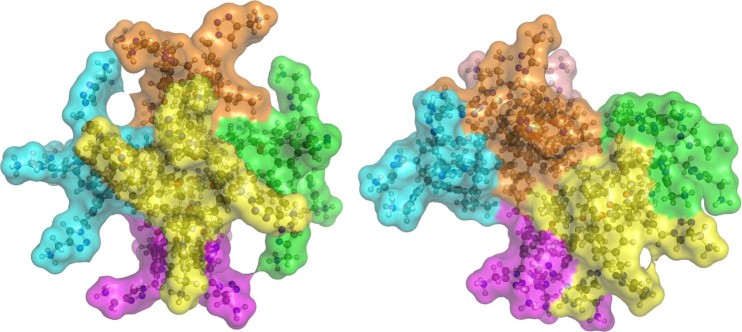
Representative MD model compatible to the SAXS data (left) compared to a model incompatible with the SAXS data (right).

Because micelles are dynamic structures it is preferrable to fit the experimental SAXS data, instead of a single best model, to an ensemble of models, in our case optimized with the Ensemble Optimization Method (EOM)^19^. The discrepancy of the scattering pattern between the ensemble representing the whole trajectory vs the more symmetric hexagonal structures with the best fit to the data is better understood if one looks at the different structures obtained through the course of the simulation (Fig. 5).

**Figure 6:**
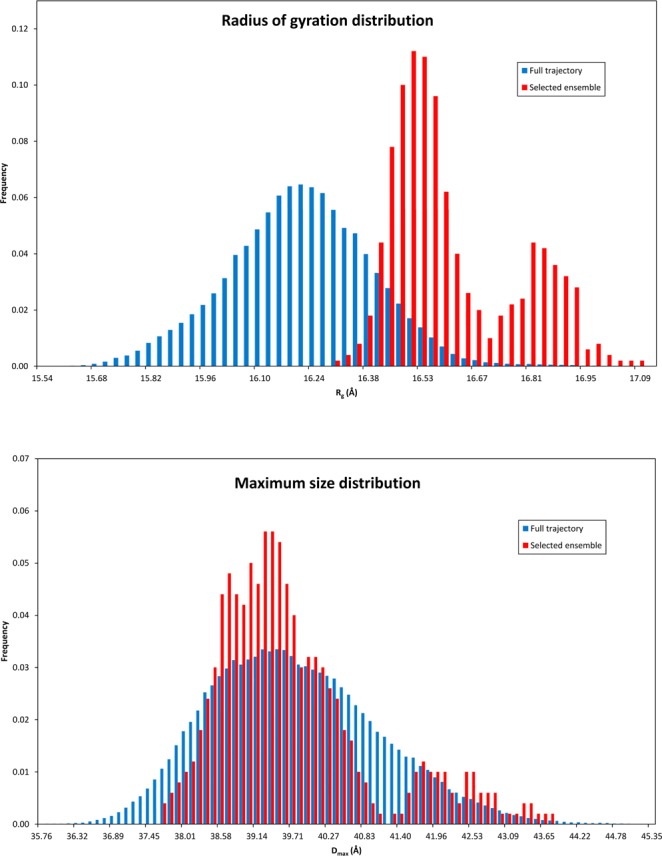
Radius of gyration and maximum particle size distribution comparison between the models obtained from a full trajectory and the ones most compatible with the experimental SAXS data.

The significant differences between all snapshots of the MD simulation and the ensembles most compatible with the data is further illustrated by comparing the histograms of their radii of gyration (*R*_g_) and maximum sizes (*D*_max_) as shown in Fig. 6. The whole trajectory histograms are normally distributed for both *R*_g_ and *D*_max_ indicating a wide range of different structures. For reference, the experimentally determined values are *R*_g_ = 16.7 Å and *D*_max_ = 45 Å^20^.

For selected structures the picture is quite different. In the case of *D*_max_, the discrepancy between the two histograms is not so great with the maximum located at roughly the same point but sharper, illustrating the shape monodispersity of the particle. The *R*_g_ histograms on the other hand are fundamentally different with the one for the selected ensemble being concetrated only at the higher values of *R*_g_. These two points lead to an interesting observation pertaining to the structure of our micelle. The structure is quite compact and symmetric and the higher electron density moieties of the micelle are located as far away from the center of mass as possible. This is facilitated both by the relative extension of the headgroups as well as keeping a very spherical shape. Indeed all micelles have higher density on their headgroups than the tails which is the main reason that one is able to model standard spherical micelles with hollow sphere models. The combination of high *R*_g_ and average *D*_max_ is a further testament to the very high symmetry of our particle, since the sphere is the most effective shape to increase the *R*_g_ while keeping the *D*_max_ small. The reason why in both distributions there is a second peak is because the selected ensemble contains some larger structures (still highly symmetric ones), distinct from the majority of structures, that are important in order to have agreement with the radius of gyration of the experimental data. Interestingly, while the experimentally determined *R*_g_ is in agreement with the average one from the selected models, *D*_max_ is clearly larger although calculating the *D*_max_ of the calculated intensities gives similarly overestimated values (possibly because of the strong hydration of the micelles).

Since it is clear that the selected subset of structures is a much better representation of the experimental SAXS data, all subsequent structural analysis and discussion refers to this and not the whole trajectory.

### Effect of ions and calix[4]arene ring conformation

**Figure 7:**
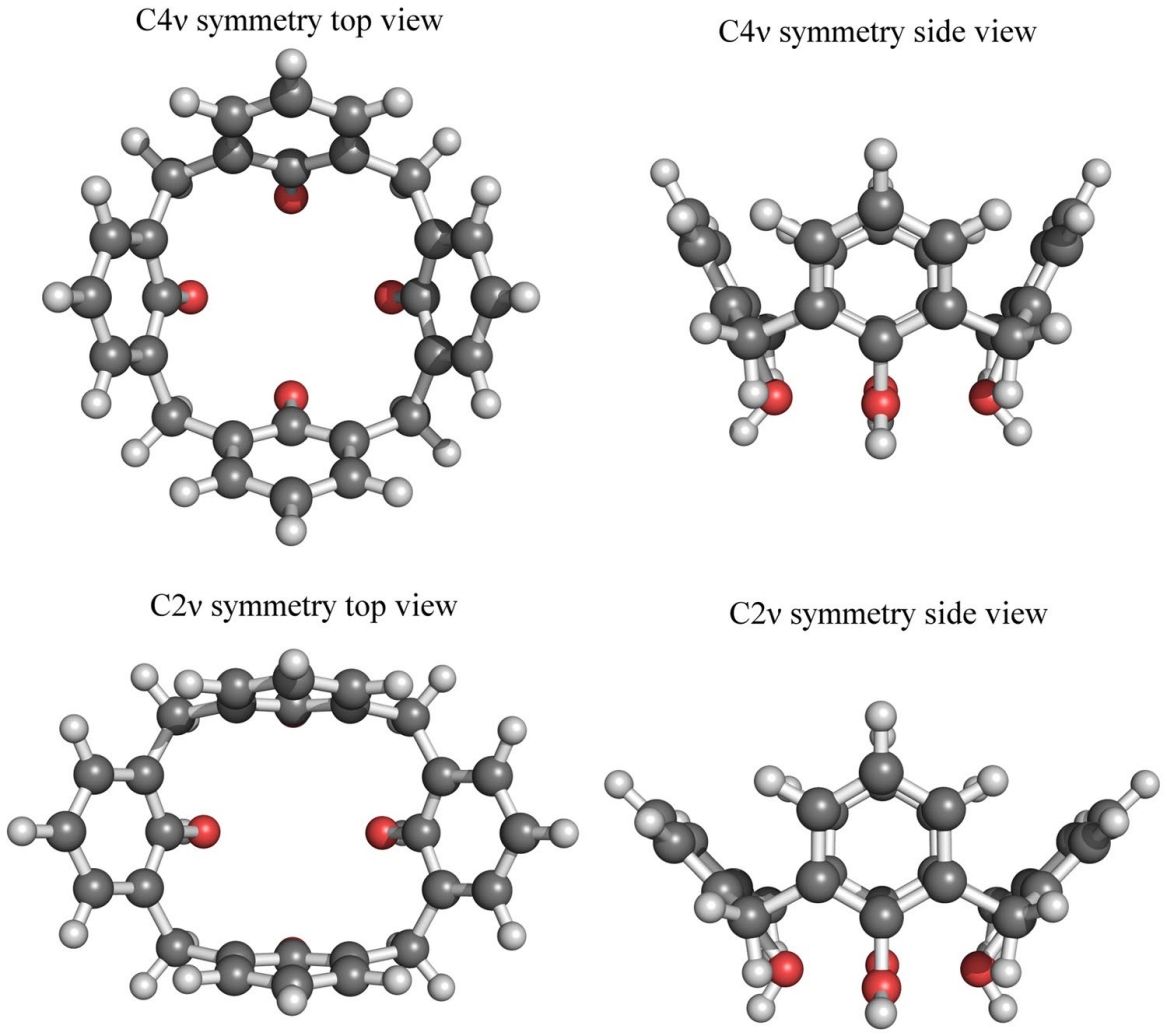
Comparison of the two possible conformations of the calix[4]arene ring.

In our previous article^5^ we have discussed the ability of the calix[4]arene ring to adopt two distinct conformations with C4ν or C2ν symmetry^21^. In the C4ν structure the four aromatic rings are equally tilted along the methylene bonds (Fig. 7 top pane). The C2ν structure has two opposite aromatic rings almost parallel to each other while the planes of the other two form an almost right angle (Fig. 7 bottom pane). C4ν is underrepresented in the crystal state and thus less stable than the C2ν one, in solution they are able to interconvert^22^. Interestingly, the coordination of sodium or potassium cations the oxygens of the lower rim of the calix[4]arene ring makes the C4ν conformation more energetically favorable because of the shielding of the electrostatic repulsions among the partially negatively charged oxygen atoms^21^. Among alkaline metals, sodium has the highest binding affinity toward the calix[4]arene derivatives^23^.

We had assumed that the sodium cation was potentially used to coordinate the oxygens of the calix[4]arene ring of PACaL3 and fix it in C4ν symmetry but the results of the MD simulations have shown that such a possibility is in fact highly unlikely. Although it was possible to fix the sodium ion in the ring by manipulating the partial charges (without this the electrostatic interactions are not strong enough and the sodium will quickly escape the calix[4]arene ring) of the molecule, any effort to model the micelle under these conditions was fruitless. The micelle disintegrates very quickly after the beginning of the simulations. Due the fact that PACaL3 is very positively charged (+4) at low pH, bringing the total net charge of the micelle to +24, adding yet one more charge to each molecule, especially close to the core of the micelle, seriously destabilizes it. Nevertheless, it is clear from the experimental results^5^ that sodium cations are important. We have to reach the conclusion that this is not due to specific interactions but rather because of the effect it has on the solvent which unfortunately we are not able to accurately assess due to the limitations of MD simulations. This may be the reason why there is an effect of smoothing the first minimum when we measured SAXS data with lithium or potassium but otherwise the SAXS patterns look very similar.

Chlorine ions are interacting much more directly with the micelle since they are needed to neutralize the charge of the heavily charged headgroups. Unlike the stable ion bridges reported by others^3^ we were not able to observe such a behavior since the chlorine anions interact only transiently with the headgroups and rarely form bridges between the headgroups of different molecules (in fact, in the rare case that a bridge is indeed formed, it is between the headgroups of the same molecule). Thus, in our case we have to assume that the role of chlorine is simply to screen the high positive charge of the micelle.

### Mobility of the building block of calix[4]arene within the micelle

**Figure 8:**
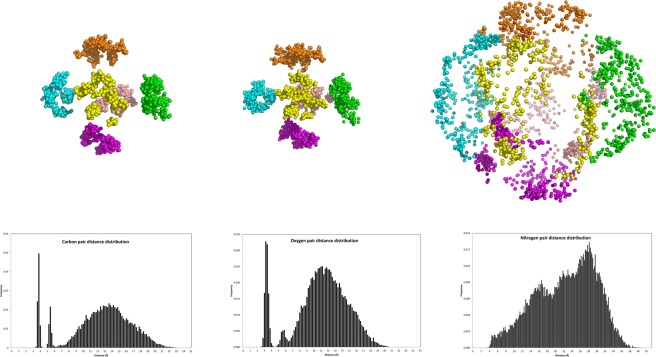
Topology of the calix[4]arene ring carbons and oxygens and the amine group nitrogens over the selected structures (top, each colour represents atoms belonging to the same PACaL3 molecule) and pair distance distribution of the respective atoms.

One interesting question is how mobile is the calix[4]arene moiety within the micelle. In order to achieve this we used the position of oxygens and carbons of the lower rim of the calix[4]arene ring as references. We have already established that the calix[4]arene ring adopts a C2ν conformation since no sodium is present. By evaluating the average pair distance distribution of structures in the selected ensemble one is able to make some interesting observations. Figure 8 shows the topology of the calix[4]arene ring carbon and oxygens and the amine nitrogens and the corresponding pair distance distributions. It is evident that the carbon and oxygen atoms of each molecule are positioned in a clearly defined and small space. The first two sharp peaks of the pair distribution histogram correspond to the distances within the same molecule while the third peak corresponds to the distances of atoms belonging to different molecules. The range of distances is from ~6.5 to ~20 Å with the peak position at ~12 Å. The reason of the Gaussian-like shape of the distribution is that distances between all the oxygens within each molecule are considered. In contrast, the much more mobile nitrogen atoms are distributed more evenly across the surface of the micelle.

The fact that the oxygen and aromatic carbon atoms show so limited mobility shows that the same applies to the whole calix[4]arene moiety. The calix[4]arene rings, in contrast to the head and tail groups, are fixed in place in very specific positions within the micelle (each roughly occupying the face of a cube) probably facilitated by numerous π-π interactions between the aromatic rings and are the main building blocks of the spherical shape and the main reason behind the high monodispersity of the micelle.

## Discussion

From the above results it is evident that MD simulations in combination with SAXS can be very valuable in determining the structure of molecules or supramolecular assemblies. The MD force fields are of course simplifications of the actual interactions that take place and thus the obtained results are not always compatible with the experiment. Even then, an MD simulation run provides the opportunity to the molecule to explore a large conformational space thus providing us with a big pool of potentially useful and realistic structures. SAXS is a great filter of this pool of structures and can be applied to a variety of different systems^19^. This approach is more desirable than the usual sphere, ab initio, coarse grain or rigid body models because it can provide information on the interactions at the atomic level by studying the MD snapshots that are compatible with the experimental SAXS data. In the case of dynamically moving micelles consisting of closely packed surfactant molecules MD may be the only available tool providing the most realistic atomic level information.

In the case of the low pH spherical hexameric micelles of PACaL3 it is clear that the micelles are rigid, symmetrical and with limited anisometry, with more elongated structures rarely occurring in the dynamic movement of the micelle. It was also shown that the ions in the solution, while important for the stability of the micelle do not interact in a specific way with the micelle. The chlorine anions act just as a neutralizing agent of the very positively charged micelle, while sodium cations probably only affect the general structure of the solvent in a way that is inaccessible by the current MD force fields. The calix[4]arene ring is found almost exclusively in the C2ν conformation. Finally, the position of the oxygens of the calix[4]arene ring changes little during the simulation, indicating that the calix[4]arene moieties are very accurately positioned in the micelle and don’t move much, unlike the headgroups and the tails.

In conclusion, the size and geometry of the molecule is very favorable for a platonic micelle with aggregation number 6.The rigidity of the main building block of calix [4]arene further reinforces the monodispersity and spherical shape of our micelle. We think it is not a coincidence that both in our case and in the case of previously reported persistent micelles^1–3,24^ there is a bulky building block, with few degrees of freedom, connecting the headgroup with the hydrophobic tails. This leads to a rigid frame of the micelle facilitating its persistence and the actual aggregation number of the micelle is a function of the overall geometry of the lipid/surfactant with the length of the tail and the size and type of the headgroup determining the number of molecules that comprise the micelle.

## Supplementary information

Supplementary_information
