# Supplementary material for: Structural analysis of a calix[4]arene-based Platonic Micelle
Source: Sci Rep. 2019 Feb 13;9:1982. doi: 10.1038/s41598-018-38280-1 (PMC6374510; doi:10.1038/s41598-018-38280-1)
Supplement: Supplementary file 1 — Supplementary_information [file 41598_2018_38280_MOESM1_ESM.pdf]

Supplementary information

Structural analysis of a calix[4]arene-based Platonic Micelle

*Efstratios Mylonas [1,2,3], Naoto Yagi[1], Shota Fujii[2], Kodai Ikesue[4], Tomoya Ueda[4], Hideaki Moriyama[4], Yusuke Sanada[2,5], Kazuya Uezu[2], Kazuo Sakurai[2,5],Tadashi Okobira[4]**

[1]Japan Synchrotron Radiation Research Institute (JASRI/SPring-8), 1-1-1, Kouto, Sayo-cho, Sayo-gun, Hyogo 679-5198, Japan

[2]Department of Chemical Processes and Environments, Faculty of Environmental Engineering, The University of Kitakyushu, 1-1, Hibikino, Wakamatsu-ku, Kitakyushu 808-0135, Japan

[3]Present address: Institute of Molecular Biology and Biotechnology, 70013 Heraklion, Crete, Greece.

[4]Department of Creative Engineering, National Institute of Technology, Ariake College 150 Higashihagio, Omuta, Fukuoka 836-8585, Japan

[5]Structural Materials Science Laboratory SPring-8 Center, RIKEN Harima Institute Research, 1-1-1 Kouto, Sayo, Sayo, Hyogo 679-5148, Japan

*Corresponding Author: Dr Tadashi Okobira, Department of Creative Engineering, Ariake KOSEN, Fukuoka, Japan.

Email address: okobira@ariake-nct.ac.jp

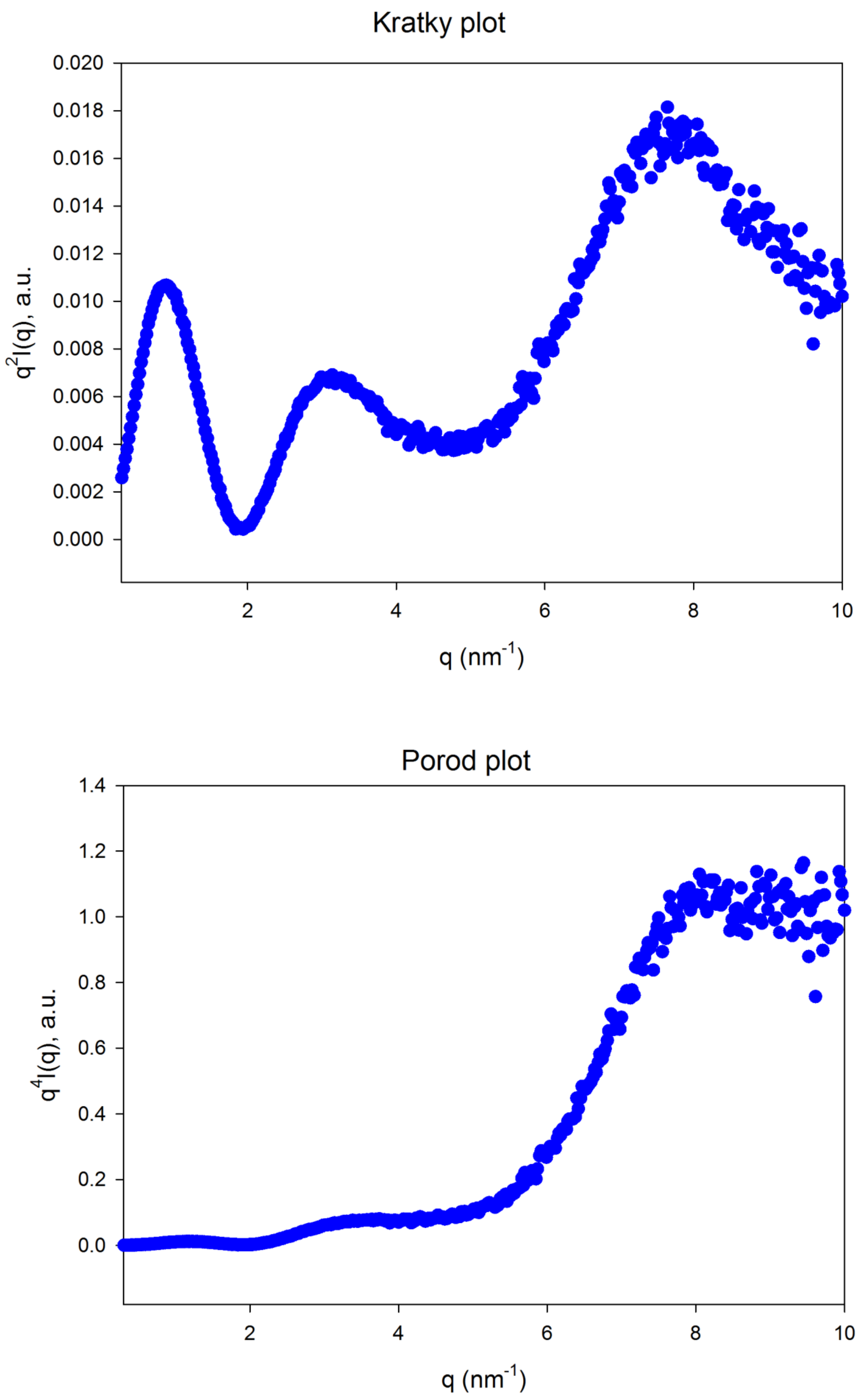

Figure S1. Kratky and Porod plots of the PACaL3 micelle

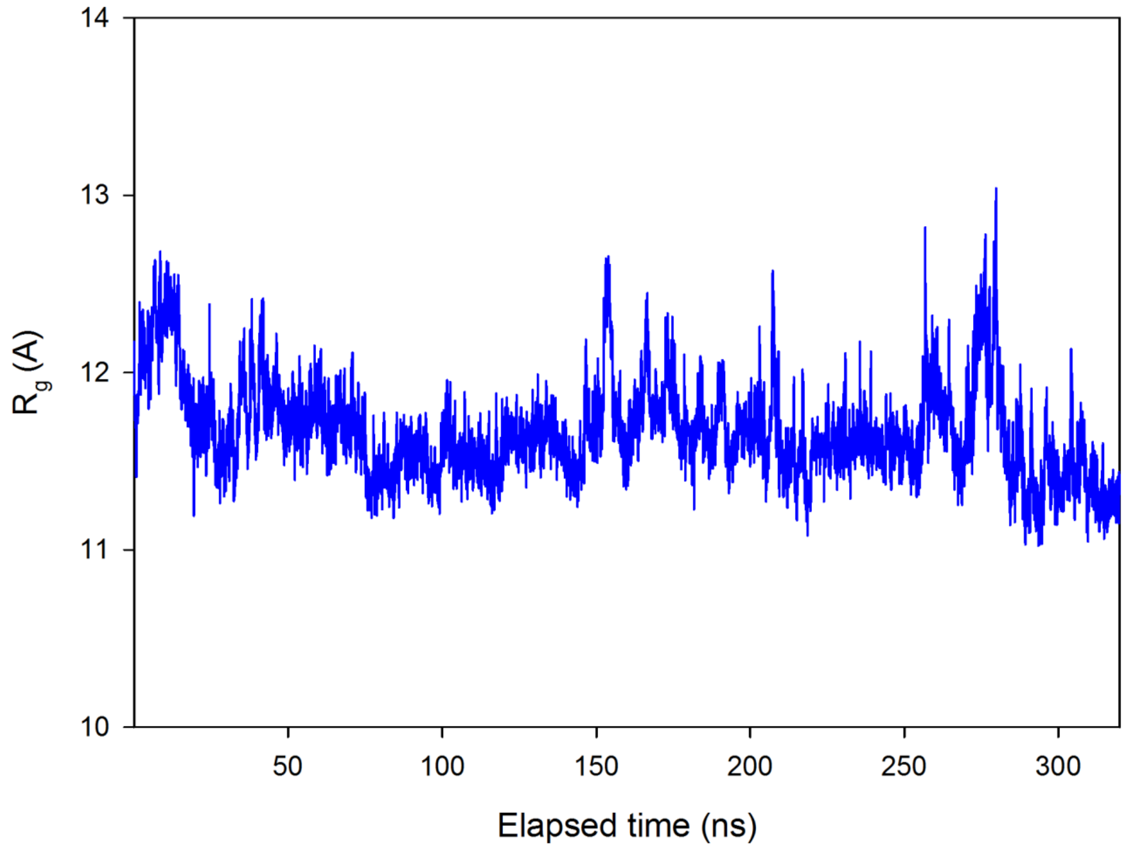

Figure S2. Temporal change of the R_g over the course of MD simulations (These values are significantly smaller than the ones estimated by CRYSOL because they are calculated with CPPTRAJ and the water shell is not accounted for).

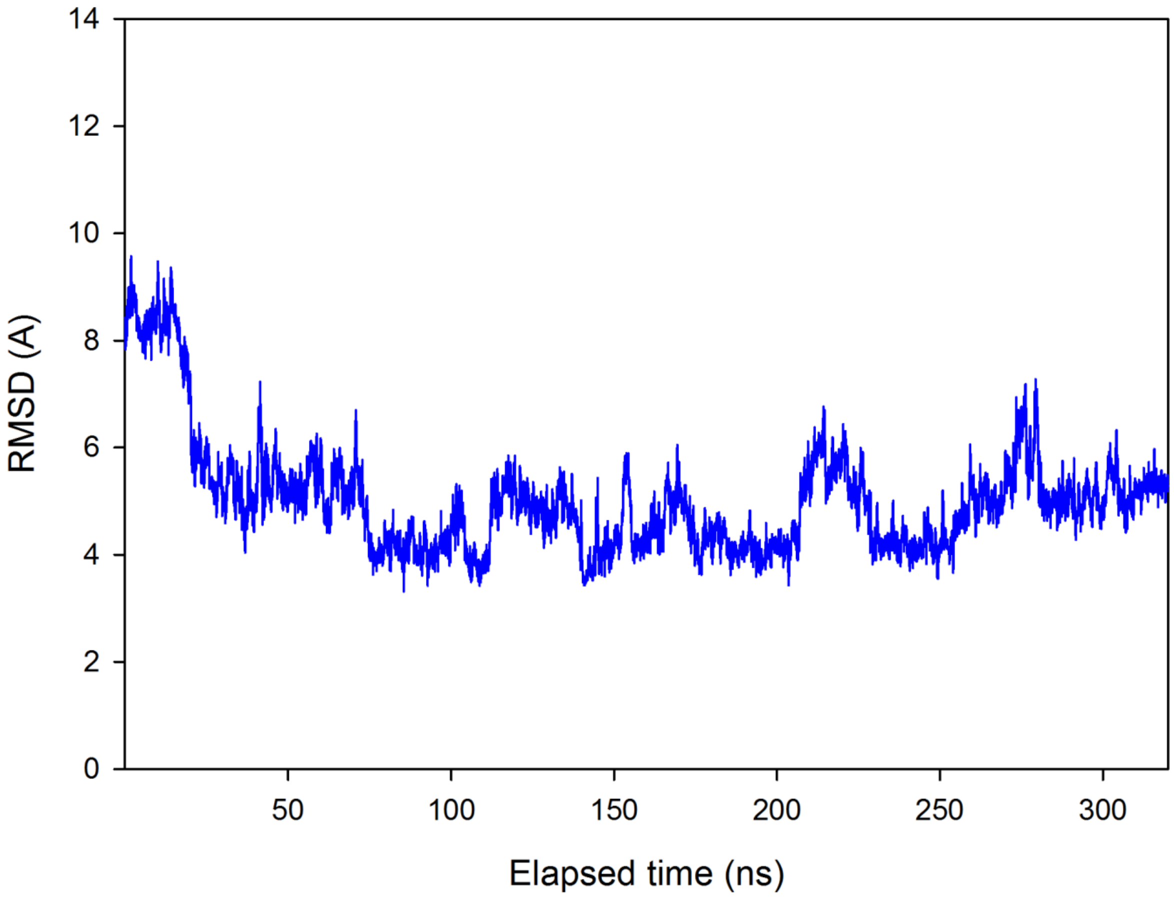

Figure S3. RMSD fluctuations of the micelle heavy atoms over the course of MD simulations compared to the average structure of the full trajectory. The values are uncharacteristically large because the micelle consists of 6 identical molecules but RMSD compares deviations only between atoms with the same identifiers.

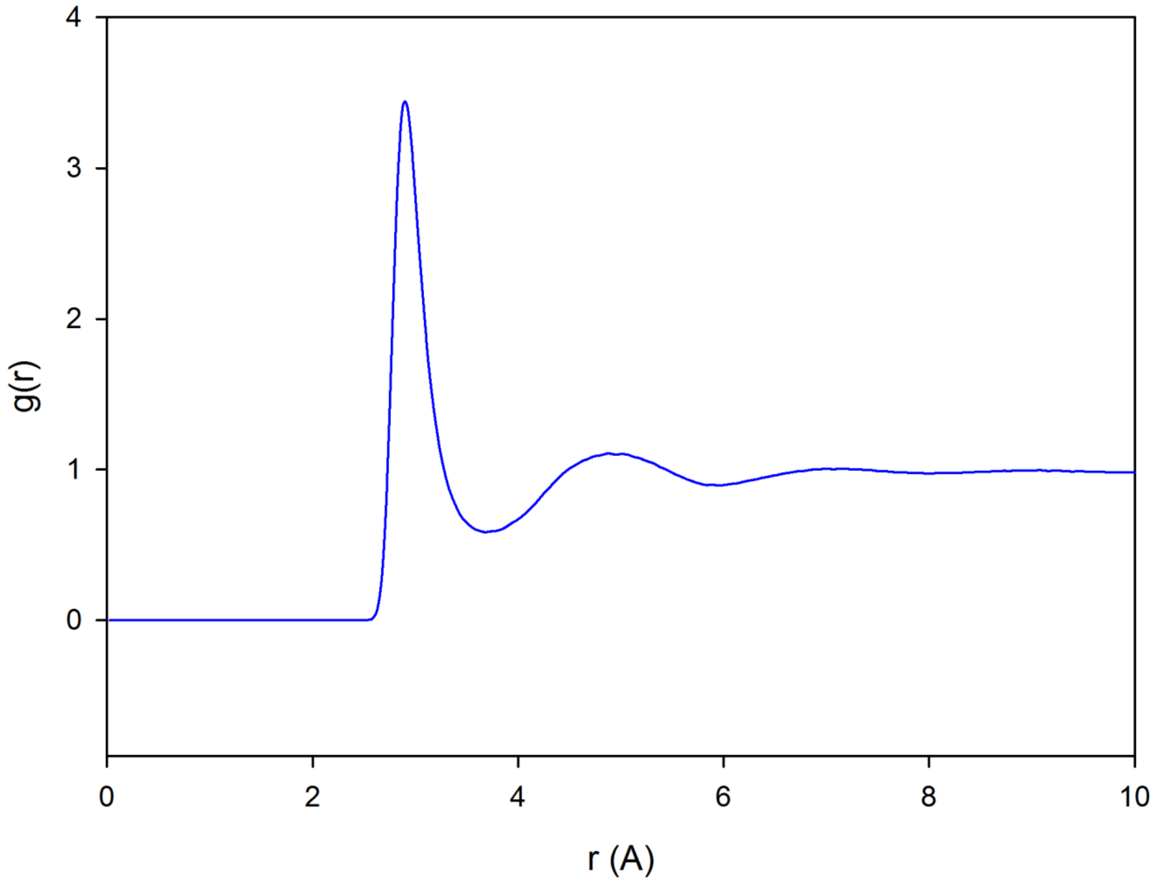

Figure S4. RDF of water oxygens around the amine group nitrogen of the PACaL3 surfactant.

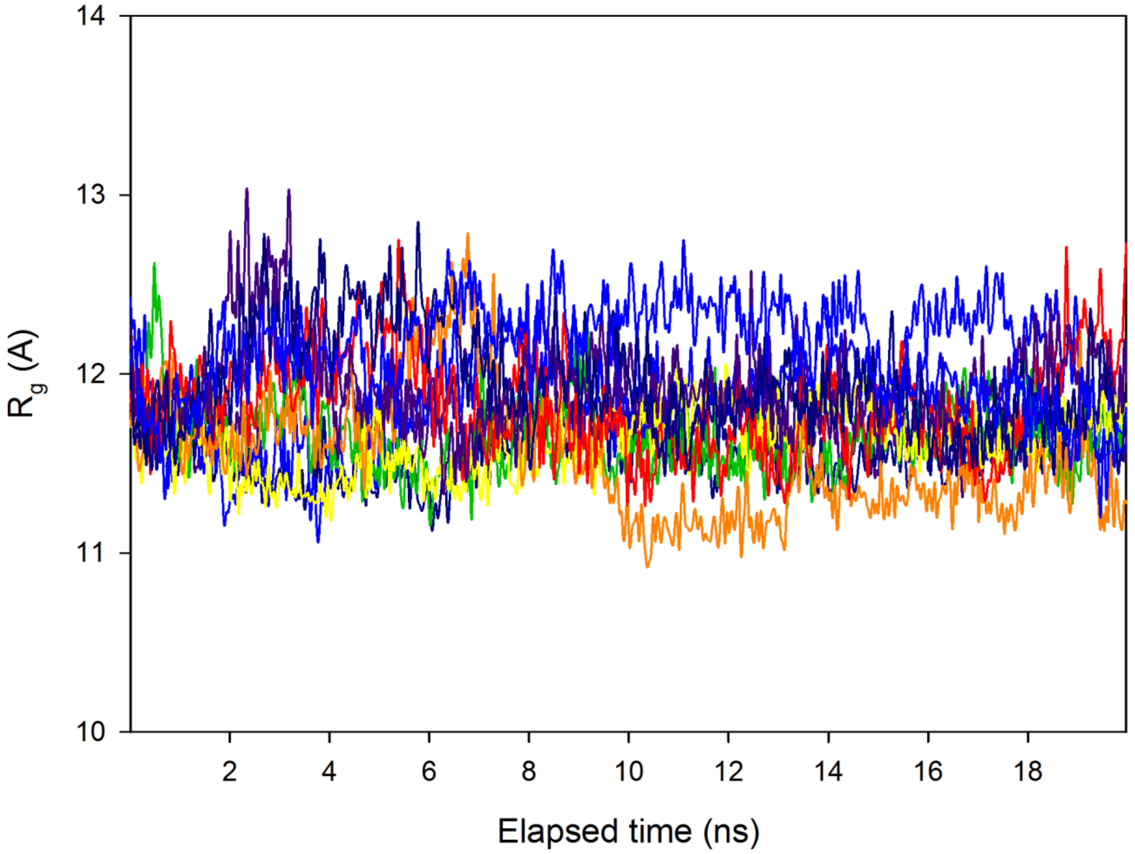

Figure S5. Temporal change of the R_g over the course of 10 MD simulations with two different starting structures. No significant differences are observed between the simulations.
